# *Pueraria thomsonii* Benth.: a high-biomass plant for cadmium phytoremediation with high accumulation in stems

**DOI:** 10.3389/fpls.2025.1693532

**Published:** 2025-11-18

**Authors:** Xing Li, Xiaqin Luo, Yusheng Zhang, Lijun Zhou, Lin Wu, Binqiang Wang, Shaolang He, Shangshu Huang, Yongming Sun, Weisheng Lv, Xiaobing Lin

**Affiliations:** 1 National Engineering and Technology Research Center for Red Soil Improvement/Jiangxi Institute of Red Soil and Germplasm Resources/Key Laboratory of Arable Land Improvement and Quality Improvement of Jiangxi Province, Nanchang, China; 2 Modern College of Agriculture and Forestry Engineering, Ganzhou Polytechnic, Ganzhou, China

**Keywords:** bioaccumulation, biomass, heavy metals, phytoremediation, *Pueraria thomsonii*

## Abstract

Heavy metal contamination in farmland soils threatens food security and human health. Phytoremediation provides a sustainable strategy, with *Pueraria thomsonii* emerging as a promising candidate due to its high-biomass production. However, its metal accumulation dynamics remain uncharacterized. Therefore, a 3-year field experiment was conducted to investigate biomass production, the accumulation of heavy metals (Cd, Cr, As, and Pb) across growth stages, and the bioconcentration factor (BCF), translocation factor (TF), and removal rate in *P. thomsonii*. Results showed that *P. thomsonii* produced a total biomass of 10.81–21.22 t ha^−1^, accumulating 76.95–170.61 (Cd), 30.80–43.28 (Cr), 7.12–9.09 (As), and 18.16–39.30 (Pb) g ha^−1^ during 2020–2022. Stem Cd accumulation exceeded root tubers and leaves by 45%–205% at maturity, while leaf Cr, As, and Pb accumulation was higher than root tubers and stems by 149%–868%. Cd-BCF values >1 in stems and leaves indicated efficient bioaccumulation, contrasting with Cr-, As-, and Pb-BCF <1. Stem and leaf TF values of Cd, Cr, As, and Pb consistently exceeded >1 except for stem Cr TF in 2022. On average, in the 3-year experimental period, the removal rate of Cd surpassed Cr, As, and Pb by 94%–95%. Soil properties (organic matter, available N) significantly influenced BCF, while available N modulated TF. These findings suggest that the combination of high-biomass production and elevated Cd accumulation in stems positions *P. thomsonii* as a promising candidate for high-biomass phytoremediation of Cd-contaminated farmland soils.

## 1 Introduction

Industrial waste-derived heavy metal pollution in soil represents a critical global environmental challenge. Mining and metallurgical activities produce large volumes of hazardous waste, which, under conditions of poor waste management and inadequate pollution control, readily migrate into soils and ecosystems via multiple pathways (Liu et al., 2019; Xie et al., 2023). A survey bulletin has revealed that 19.4% of China’s farmland soil was affected by various pollutants (Chen et al., 2014). Among these contaminants, cadmium (Cd), chromium (Cr), arsenic (As), and lead (Pb) were identified as the critical inorganic contaminants, collectively accounting for 7.0%, 1.1%, 2.7%, and 1.5% of the total soil pollution, respectively (Zhao et al., 2015). Soil contamination with toxic elements constitutes a pressing ecological concern, posing profound ramifications for human health (Wang et al., 2019; Vasilachi et al., 2023). The accumulation of these heavy metals in human bodies can lead to a range of diseases, such as cancer, dermal lesions, and kidney dysfunction, via the food chain (Wang and Xia, 2015; Wang et al., 2020). It is therefore crucial to develop diverse remediation strategies that target the removal of heavy metals from polluted soils.

Phytoremediation exhibits significant advantages over conventional remediation methods, providing cost-effectiveness, broad applicability, and efficacy in heavy metal-contaminated soil remediation (Awa and Hadibarata, 2020). However, prominent hyperaccumulator species currently employed in phytoremediation face significant limitations, including specialized agronomic requirements and low biological returns (Lin et al., 2014). Consequently, identifying metal-accumulating plants with high biomass and yield, substantial economic valorization potential, and adaptability to subtropical southern China’s cultivation conditions becomes imperative. Researchers have extensively documented interspecific variations in heavy metal uptake and accumulation among plant species. While certain plants like rice and barley show pronounced Cd and As accumulation, others such as wheat and corn exhibit relatively lower uptake (Abedi et al., 2022; Khan et al., 2023). Documented hyperaccumulators, including *Pteris vittata* (for As and Cr) and *Sedum alfredii* (for Pb and Cd), demonstrate remarkable extraction efficiency (Yan et al., 2020). However, their practical application in large-scale farmland remediation is often constrained by limited biomass, agronomic requirements, and low economic returns. This limitation has shifted the research focus toward identifying crops that combine high-biomass production with substantial metal accumulation capacity, aiming to achieve simultaneous soil remediation and economic benefits (Dou et al., 2022). Crucially, *Pueraria thomsonii* Benth., valued for its dual medicinal and edible applications, produces substantial biomass, positioning it as a promising hyperaccumulator candidate for phytoremediation (Liu et al., 2021; Xu et al., 2025).

*Pueraria thomsonii*, a leguminous plant extensively cultivated in China, primarily grows in the provinces of Guangxi, Jiangxi, Hubei, Hunan, Sichuan, and Yunnan (Liu et al., 2021). Its root tuber serves as a source for developing therapeutic agents and dietary supplements due to high concentrations of bioactive isoflavones, notably puerarin, daidzin, and rutin (Liu et al., 2015). The abundant root tuber starch exhibits significant potential for food processing and industrial applications, further documenting elevated selenium and strontium in root tubers during the expansion stage, contrasting with trace Cd, Cr, As, and Pb (Liu et al., 2021). However, the dynamics of heavy metal accumulation, translocation, and tissue partitioning across growth stages remain uncharacterized. Understanding these mechanisms is critical for evaluating its remediation potential, managing the risk of Cd transfer to edible parts, and ultimately developing it into a viable phytoremediation resource. Therefore, we conducted a 3-year field experiment to investigate biomass production, the accumulation of heavy metals (Cd, Cr, As, and Pb) across growth stages, and the bioconcentration factor (BCF), translocation factor (TF), and removal rate for *P. thomsonii* in different tissues. The primary objective was to assess its potential as a phytoremediation candidate.

## 2 Materials and methods

### 2.1 Site description and experimental design

**Figure 1:**
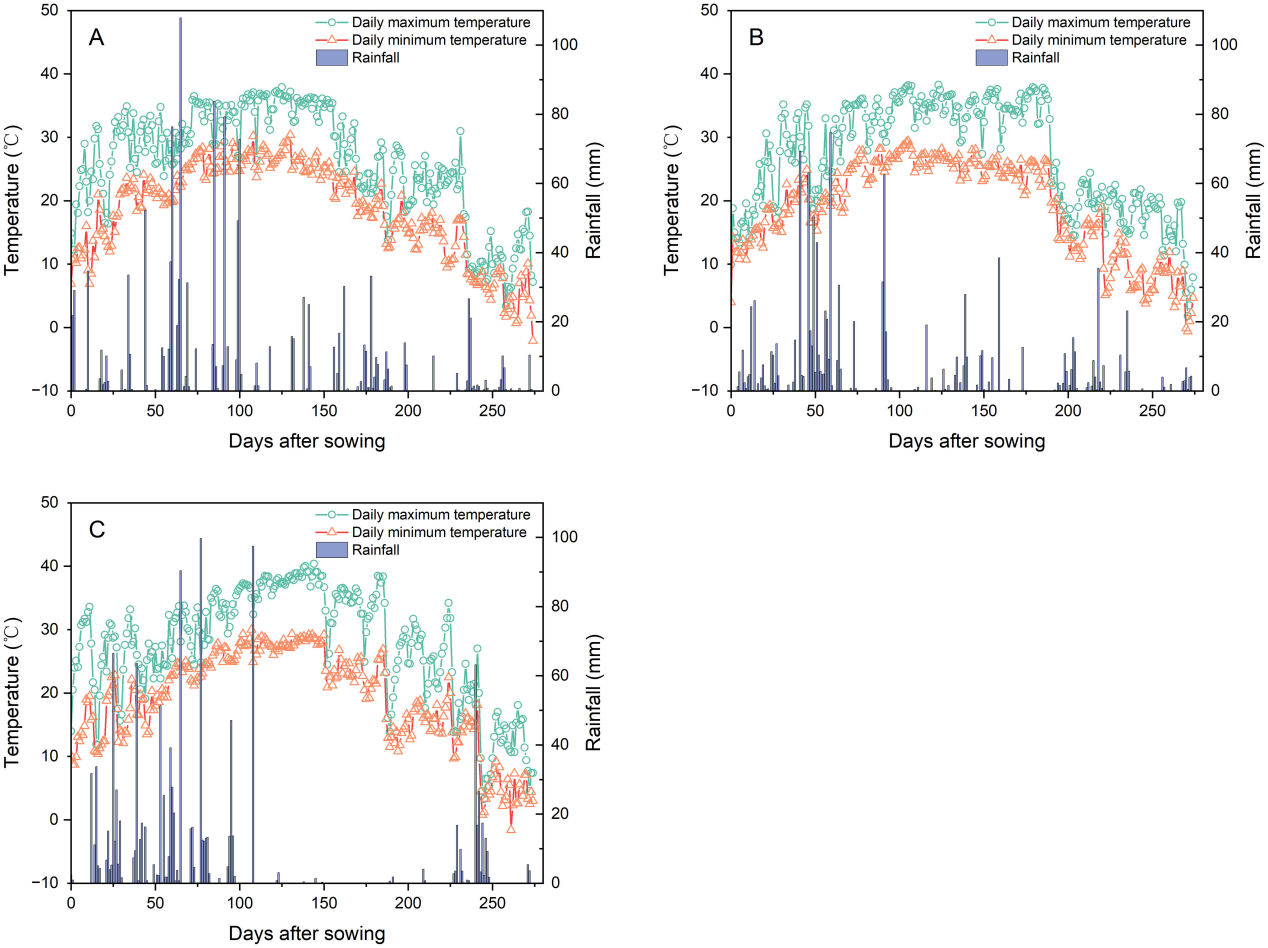
Daily temperatures (maximum/minimum) and precipitation during the *Pueraria thomsonii* growing seasons in 2020 **(A)**, 2021 **(B)**, and 2022 **(C)**.

Field experiments were conducted from 2020 to 2022 at the research farm of Jiangxi Institute of Red Soil and Germplasm Resources (27°39′30″N, 113°42′24″E, 64 m above sea level), Jiangxi Province, China. The site features a humid subtropical monsoon climate. During the experimental growing seasons, the average maximum temperatures were 26.1°C (2020), 26.9°C (2021), and 27.6°C (2022), while the average minimum temperatures were recorded at 18.6°C, 19.0°C, and 18.9°C, respectively (**Figure 1**). Total precipitation recorded at the site over the experimental periods was 1,345 mm (2020), 1,245 mm (2021), and 1,149 mm (2022) (**Figure 1**). Prior to annual experiment initiation, composite soil samples (0–20 cm depth) were collected. The soil properties are presented in **Table 1**.

**Table 1:** Soil properties before land preparation during 2020–2022.

Year	pH	Organic matter	Available N	Available P	Available K	Soil Cd content	Soil Cr content	Soil As content	Soil Pb content
		(g kg^−1^)	(mg kg^−1^)	(mg kg^−1^)	(mg kg^−1^)	(mg kg^−1^)	(mg kg^−1^)	(mg kg^−1^)	(mg kg^−1^)
2020	5.64	46.15	242	15.92	162	15.63	86.36	17.73	76.79
2021	5.87	47.74	146	35.37	161	14.47	60.13	15.05	50.73
2022	5.84	33.61	151	50.99	67	11.64	62.91	11.06	57.10

Data are the means of 12 replications for each year.

A cultivated *P. thomsonii* variety, Gange 1, was planted annually throughout the 3-year study period. Bred by Jiangxi Institute of Red Soil and Germplasm Resources, this variety was characterized by short growth duration, high yield, and high starch content, thus achieving extensive cultivation. The experiment employed a randomized block design with 12 replications per year, using plots measuring 30 m². The seedlings were transplanted into the field on April 1 at a density of 27,000 plants ha^−1^ (single seedling per hill). Harvesting occurred on December 31 of each year, resulting in a total growth duration of 275 days from transplanting to harvest. Basal fertilization consisted of 2,400 kg ha^−1^ of 45% sulfate of potash compound fertilizer (N–P_2_O_5_–K_2_O, 15–15–15). The remaining management practices were consistent with regional high-yield cultivation standards.

### 2.2 Sampling and measurements

At the seeding stage (SN), expansion stage (EP), and maturity stage (MA), five hills of plants were randomly sampled in each plot. Kudzu plants were separated into roots, stems, and leaves at the seeding stage and separated into root tubers, stems, and leaves at the expansion and maturity stages. These components were then dried in an oven at 70°C to a constant weight to determine biomass production. The oven-dried samples at the three stages were ground into a powder. Approximately 0.1 g of each powder was used to determine the content of heavy metals (Cd, Cr, As, and Pb) using ICP-MS (Agilent 7900, USA) according to the method described by Wan et al (Wan et al., 2019). The operating conditions for ICP-MS were set according to the method of Xie et al (Xie et al., 2020), as detailed in **Table 2**. The BCF was calculated as the heavy metal content in kudzu tissues (including root tuber, stem, and leaf) to that in soil. The TF was calculated as the heavy metal content in stems or leaves to that in the root tuber. The removal rate was calculated as heavy metal accumulation at MA in kudzu per hectare to heavy metal accumulation in surface soil per hectare, with a soil depth of 20 cm and a bulk density of 1.2 g cm^−3^. The heavy metal accumulation was determined by multiplying the metal content in plant tissues by the corresponding biomass production at each growth stage.

**Table 2:** The 7900 ICP-MS operating parameters.

Parameter	Value	Parameter	Value
Radio frequency power	1.45 V	Carrier gas flow rate	0.85 L/min
Orifice of skimmer cone	0.4 mm	Mixed gas flow rate	0.28 L/min
Orifice of sampling cone	1.0 mm	Plasma gas flow rate	15.0 L/min
Sample depth	7.0 mm	Auxiliary gas flow rate	1.0 L/min
Sample uptake amount	0.4 mL/min	Helium flow rate	5 mL/min
Sample uptake flow rate	0.1 r/s	Spray chamber temperature	2°C

Fresh root tubers were thoroughly washed, peeled, and diced. The root pieces were then homogenized and placed into a 200-mesh filter bag. The bag was kneaded in distilled water for 10 min to extract the starch. The resulting slurry was filtered, and the filtrate was allowed to settle for 3 h, after which the supernatant was discarded. This washing and sedimentation were repeated three times. The recovered starch was dried at 45°C until the moisture content reached approximately 10% (w/w), ground into a fine powder using a 100-mesh sieve, and stored in a desiccator with silica gel for subsequent analysis.

### 2.3 Statistical analysis

Data analysis was performed using Statistix 8.0 (Tallahassee, FL, USA), including one-way analysis of variance (ANOVA) with the LSD_0.05_ and Pearson’s correlation analysis. Figures were created using the Origin software (Version 8.6).

## 3 Results

### 3.1 Biomass production

Total and root tuber biomass of *P. thomsonii* increased significantly from the SN to the MA stage across all three experimental years (**Table 3**). Specifically, the total biomass at EP and SN was substantially lower than at MA, with reductions of 59% and 98% in 2020, 61% and 99% in 2021, and 75% and 98% in 2022, respectively. A similar trend was observed for root tuber biomass. At MA, the total biomass ranged from 10.81 to 21.22 t ha^−1^, with the biomass of root tubers, stems, and leaves ranging from 6.13 to 13.59, 3.40 to 5.05, and 0.97 to 2.58 t ha^−1^, respectively, from 2020 to 2022.

**Table 3:** Biomass production in *Pueraria thomsonii* tissues at different growth stages during 2020–2022.

Growth stage	2020				2021				2022			
	Root tuber (t ha^−1^)	Stem (t ha^−1^)	Leaf (t ha^−1^)	Total (t ha^−1^)	Root tuber (t ha^−1^)	Stem (t ha^−1^)	Leaf (t ha^−1^)	Total (t ha^−1^)	Root tuber (t ha^−1^)	Stem (t ha^−1^)	Leaf (t ha^−1^)	Total (t ha^−1^)
SN	0.18 ± 0.02c	0.15 ± 0.06c	0.24 ± 0.05b	0.57 ± 0.11c	0.13 ± 0.05c	0.14 ± 0.04b	0.16 ± 0.06b	0.43 ± 0.10c	0.14 ± 0.03c	0.17 ± 0.03c	0.21 ± 0.05c	0.52 ± 0.09c
EP	4.90 ± 2.35b	2.57 ± 0.79b	2.68 ± 0.78a	10.15 ± 3.72b	5.31 ± 1.01b	4.50 ± 1.47a	2.31 ± 1.26a	12.12 ± 2.87b	1.56 ± 0.36b	1.62 ± 0.35b	1.98 ± 0.33a	5.16 ± 0.87b
MA	11.99 ± 2.79a	3.40 ± 1.10a	2.56 ± 0.74a	17.95 ± 4.43a	13.59 ± 4.03a	5.05 ± 1.30a	2.58 ± 0.62a	21.22 ± 4.97a	6.13 ± 0.99a	3.71 ± 1.02a	0.97 ± 0.11b	10.81 ± 1.39a

Data are presented as means ± SD. Within a column, values sharing different lowercase letters differ significantly at the 0.05 probability level.

SN, seeding stage; EP, expansion stage; MA, maturity.

### 3.2 Content of heavy metals

**Figure 2:**
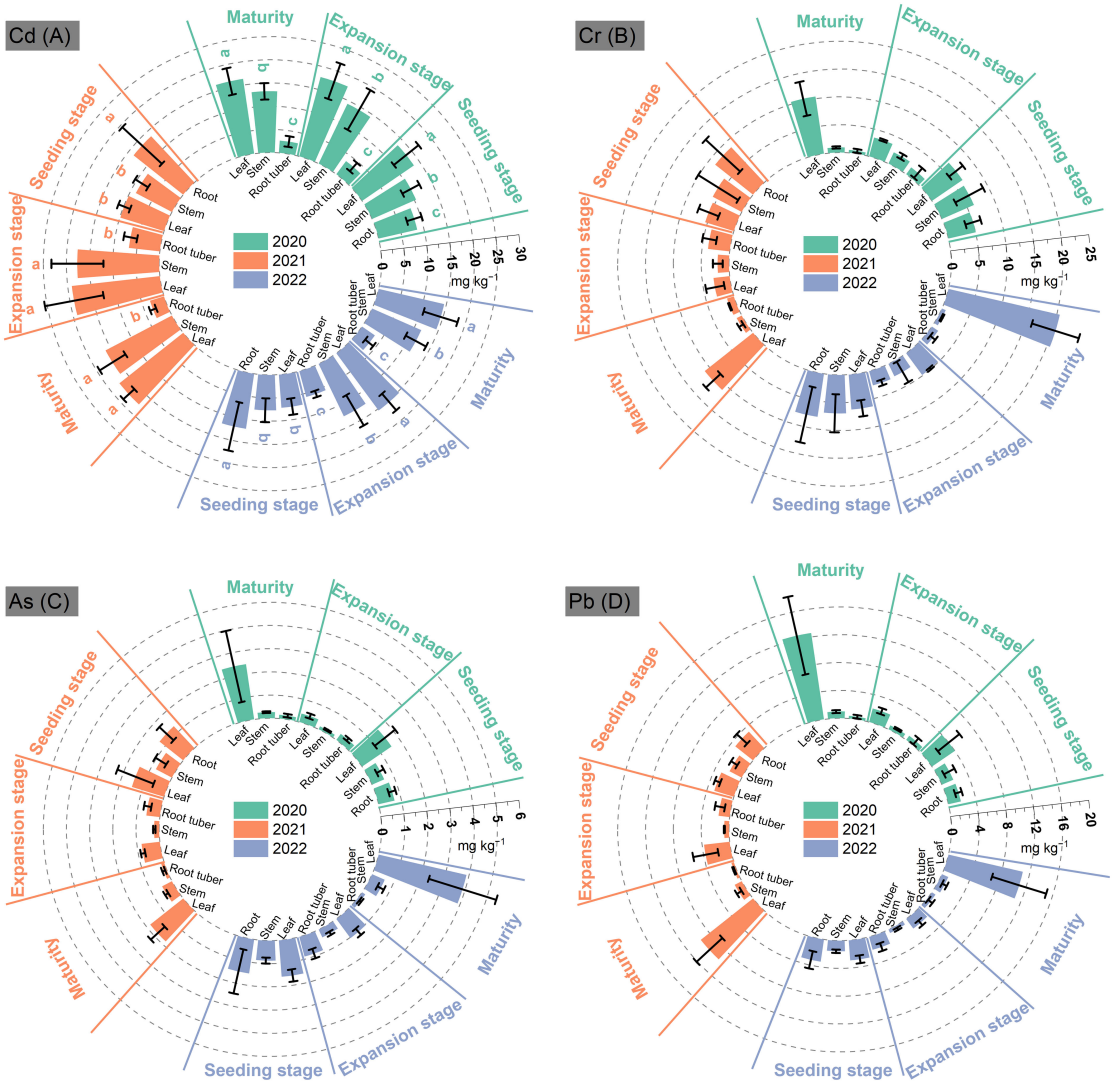
Contents of Cd **(A)**, Cr **(B)**, As **(C)**, and Pb **(D)** in *Pueraria thomsonii* tissues at different growth stages during 2020–2022. SN, seeding stage; EP, expansion stage; MA, maturity. Columns represent the means of 12 replicates with vertical bars indicating standard errors. Different lowercase letters denote significant differences at the 0.05 probability level within a year.

During the growth of *P. thomsonii*, the Cd content in root tuber showed a decreasing trend in 3 years, with the lowest at MA compared to that at EP and SN—25% and 69% in 2020, 53% and 77% in 2021, and 42% and 72% in 2022, respectively (**Figure 2A**). Stem Cd content at MA exceeded that at EP by 28%, 84%, and 51% in 2020, 2021, and 2022, respectively. In contrast, no significant differences were observed between MA and EP. Leaf Cd content at MA increased by 20%, 78%, and 102% compared to the SN in 2020, 2021, and 2022, respectively. However, no significant differences were observed between MA and EP. The Cd content in root tubers, stems, and leaves ranged from 2.65 to 3.33 mg kg^−1^, 11.56 to 16.16 mg kg^−1^, and 14.50 to 17.54 mg kg^−1^ at MA during 2022–2022, respectively.

The Cr content in root tubers and stems showed a consistent decreasing trend from SN to MA across the 3 years, with the lowest levels at MA that were 83%–90% lower than those at SN (**Figure 2B**). However, leaf Cr content first decreased and then increased, with peaking levels at MA in each year. At MA, the Cr content in *P. thomsonii* tissues was 0.52–1.34 mg kg^−1^ in root tubers, 0.73–1.01 mg kg^−1^ in stems, and 10.40–20.55 mg kg^−1^ in leaves during 2020–2022.

Root tuber As and Pb contents exhibited consistent decreasing trends from SN to MA over 3 years (**Figures 2C, D**). In contrast, stem and leaf As and Pb contents both showed initial decreases followed by increases, with stem concentrations peaking at SN and leaf concentrations peaking at MA. During 2020–2022, the As content at MA varied among tissues, with concentrations of 0.14–0.17 mg kg^−1^ in root tubers, 0.26–0.59 mg kg^−1^ in stems, and 1.81–3.89 mg kg^−1^ in leaves. Similarly, the Pb content was 0.37–0.61 mg kg^−1^ in root tubers, 0.97–1.13 mg kg^−1^ in stems, and 4.08–12.56 mg kg^−1^ in leaves.

### 3.3 Accumulation of heavy metals

**Figure 3:**
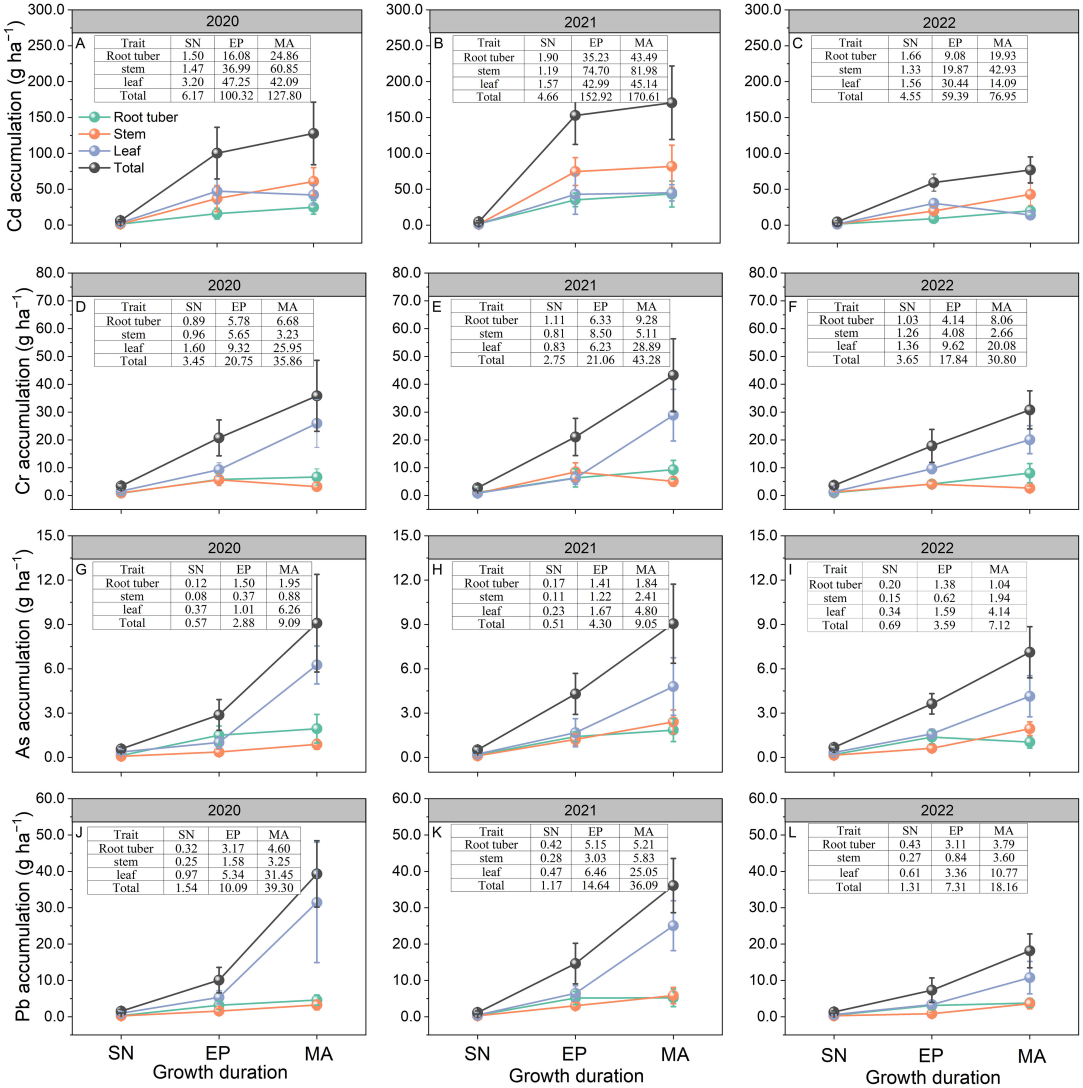
Accumulation of Cd **(A–C)**, Cr **(D–F)**, As **(G–I)**, and Pb **(J–L)** in *Pueraria thomsonii* tissues at different growth stages during 2020–2022. Data represent the means of 12 replicates with vertical bars indicating standard errors. SN, seeding stage; EP, expansion stage; MA, maturity. The data in the tables represent the data corresponding to each point in the subgraph.

**Figure 4:**
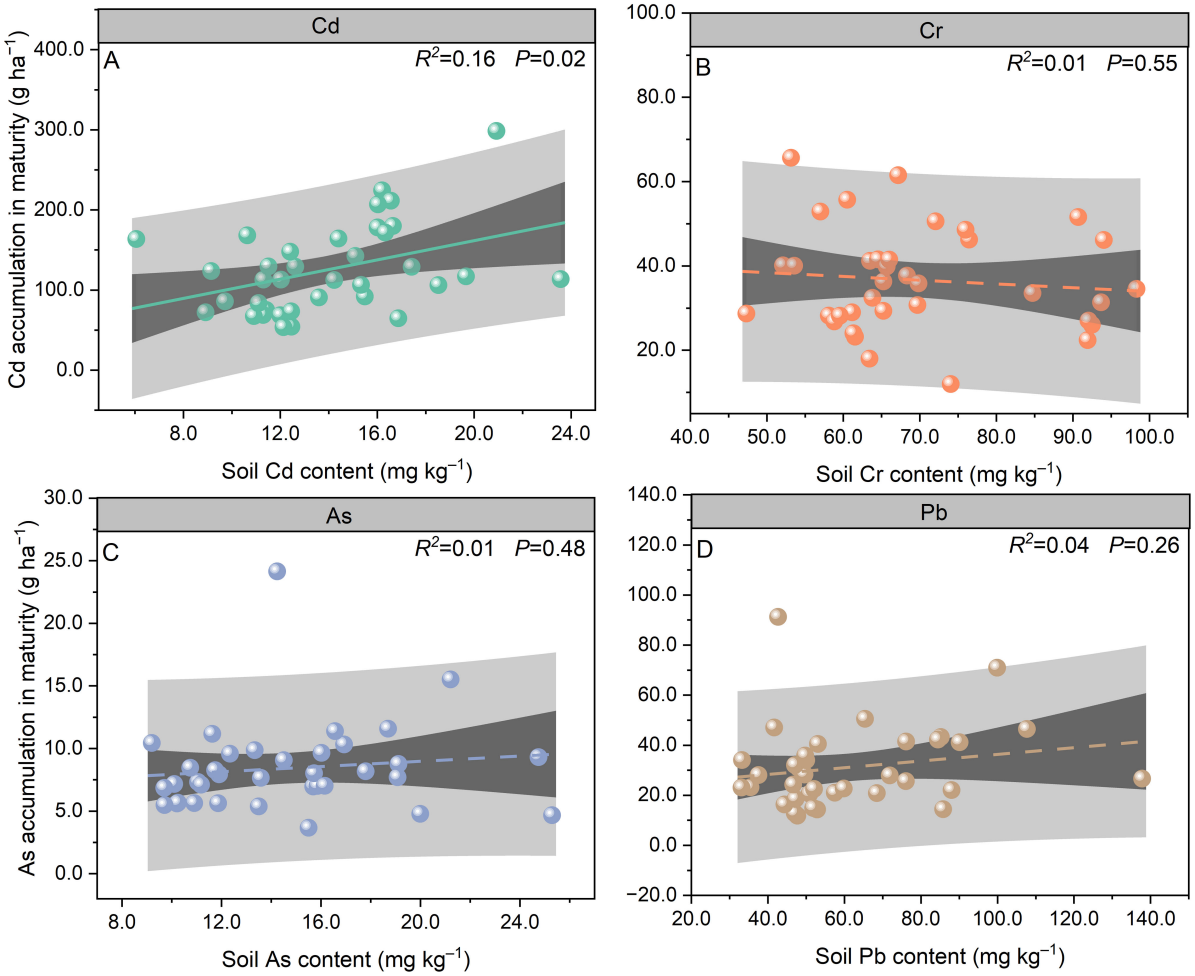
Relationships of soil Cd **(A)**, Cr **(B)**, As **(C)**, and Pb **(D)** contents to total Cd **(A)**, Cr **(B)**, As **(C)**, and Pb **(D)** accumulation in *Pueraria thomsonii* during 2020–2022 (*n* = 36). Data are the 36 replications in 3 years.

The total Cd accumulation from EP to MA was 71%, 88%, and 68% lower than the accumulation from SN to EP in 2020, 2021, and 2022, respectively (**Figures 3A–C**). Compared to stem Cd accumulation at MA, root tuber and leaf accumulation decreased by 59% and 31% in 2020, 47% and 45% in 2021, and 54% and 67% in 2022, respectively. Total Cd accumulation at MA varied from 76.95 to 170.61 g ha^−1^ during 2020–2022. Soil Cd content was significantly positively correlated with the total Cd accumulation at MA (*P* < 0.05; **Figure 4A**).

No significant differences were observed in total Cr accumulation between SN to EP and EP to MA growth periods across the 3 years (**Figures 3D–F**). In 2020 and 2021, leaf Cr accumulation from SN to EP was 54% and 76% lower, respectively, than during EP to MA. However, no significant differences were observed between these growth periods in 2022. At MA, root tuber and stem Cr accumulation decreased by 74% and 88% in 2020, 68% and 82%, and 60% and 87% in 2022, respectively, relative to leaf Cr accumulation. Total Cr accumulation at MA ranged from 30.80 to 43.28 g ha^−1^ during 2020–2022. There was no significant correlation between soil Cr content and the total Cr accumulation at MA (*P* > 0.05; **Figure 4B**).

In 2020, leaf and total As accumulation rates from SN to EP were 88% and 63% lower, respectively, than during EP to MA. However, no significant differences were observed between these growth periods for leaf and total As accumulation in 2021 and 2022 (**Figures 3G–I**). At MA, root tuber and stem As accumulation was 69% and 86%, 61% and 50%, and 75% and 53% lower than that of leaf in 2020, 2021, and 2022, respectively. Total As accumulation at MA varied from 7.12 to 9.09 g ha^−1^ during 2020–2022. No significant correlation was observed between soil As content and the total As accumulation at MA (*P* > 0.05; **Figure 4C**).

The leaf and total Pb accumulation rates from EP to MA were 83% and 71%, 68% and 37%, and 63% and 45% lower than during SN to EP in 2020, 2021, and 2022, respectively (**Figures 3J–L**). Compared to leaf Pb accumulation at MA, root tuber and stem accumulation decreased by 85% and 90% in 2020, 79% and 77% in 2021, and 65% and 67% in 2022, respectively. Total Pb accumulation in plants at MA ranged from 18.16 to 39.30 g ha^−1^ during 2020–2022. Soil Pb content was not correlated with the total Pb accumulation at MA (*P* > 0.05; **Figure 4D**).

### 3.4 Bioconcentration factor, translocation factor, and removal rate

The BCF values for Cd in root tubers ranged from 0.14 to 0.29, while stem BCF values and leaf BCF values ranged from 1.09 to 1.34 across the 3 years (**Table 4**). Compared to stems and leaves, root tuber BCF values were consistently lower by 88% and 87% in 2020, 82% and 82% in 2021, and 76% and 78% in 2022, respectively. For Cr, As, and Pb, root tuber and stem BCF values exhibited reductions of 75%–97% relative to leaf BCF values across the 3 years.

**Table 4:** Bioconcentration factors (BCFs) and translocation factors (TFs) in *Pueraria thomsonii* tissues at maturity during 2020–2022.

Metal	Year	BCF_Root tuber_	BCF_Stem_	BCF_Leaf_	TF_Stem_	TF_Leaf_
Cd	2020	0.14 ± 0.07b	1.17 ± 0.37a	1.09 ± 0.29a	9.10 ± 3.37a	9.08 ± 4.49a
	2021	0.24 ± 0.08b	1.26 ± 0.66a	1.34 ± 0.53a	5.35 ± 1.69a	5.77 ± 1.25a
	2022	0.29 ± 0.10b	1.20 ± 0.13a	1.29 ± 0.52a	4.28 ± 1.25a	4.85 ± 1.97a
Cr	2020	0.01 ± 0.01b	0.01 ± 0.01b	0.12 ± 0.03a	3.06 ± 3.08b	32.41 ± 12.68a
	2021	0.01 ± 0.01b	0.02 ± 0.01b	0.19 ± 0.04a	1.57 ± 0.8b	16.85 ± 3.55a
	2022	0.02 ± 0.01b	0.01 ± 0.01b	0.33 ± 0.08a	0.63 ± 0.30b	17.99 ± 9.11a
As	2020	0.01 ± 0.01b	0.02 ± 0.01b	0.15 ± 0.11a	1.95 ± 0.93b	19.42 ± 6.97a
	2021	0.01 ± 0.01c	0.03 ± 0.01b	0.12 ± 0.03a	3.97 ± 1.66b	15.45 ± 7.15a
	2022	0.02 ± 0.01b	0.05 ± 0.01b	0.38 ± 0.11a	3.50 ± 1.19b	28.72 ± 16.39a
Pb	2020	0.01 ± 0.01b	0.01 ± 0.01b	0.18 ± 0.11a	3.73 ± 3.03b	52.47 ± 22.51a
	2021	0.01 ± 0.01b	0.02 ± 0.01b	0.20 ± 0.07a	3.19 ± 1.23b	28.3 ± 12.02a
	2022	0.01 ± 0.01b	0.02 ± 0.01b	0.19 ± 0.06a	2.08 ± 1.28b	22.85 ± 12.34a

Data are presented as means ± SD. Within a row, values sharing different lowercase letters differ significantly at the 0.05 probability level.

The TF values for Cd in stems and leaves ranged from 4.28 to 9.10, with the mean of 6.24 for stems and 6.56 for leaves across the 3 years (**Table 4**). For Cd, no significant differences were observed between the stem TF values and leaf TF values in each year. The TF values for Cr in stems were lower than those in leaves by 91%, 91%, and 96% in 2020, 2021, and 2022, respectively. The stem TF values for As decreased by 90% (2020), 74% (2021), and 88% (2022) relative to the leaf TF values. Similarly, the stem TF values for Pb were 93%, 89%, and 91% lower than the leaf TF values in 2020, 2021, and 2022, respectively.

**Figure 5:**
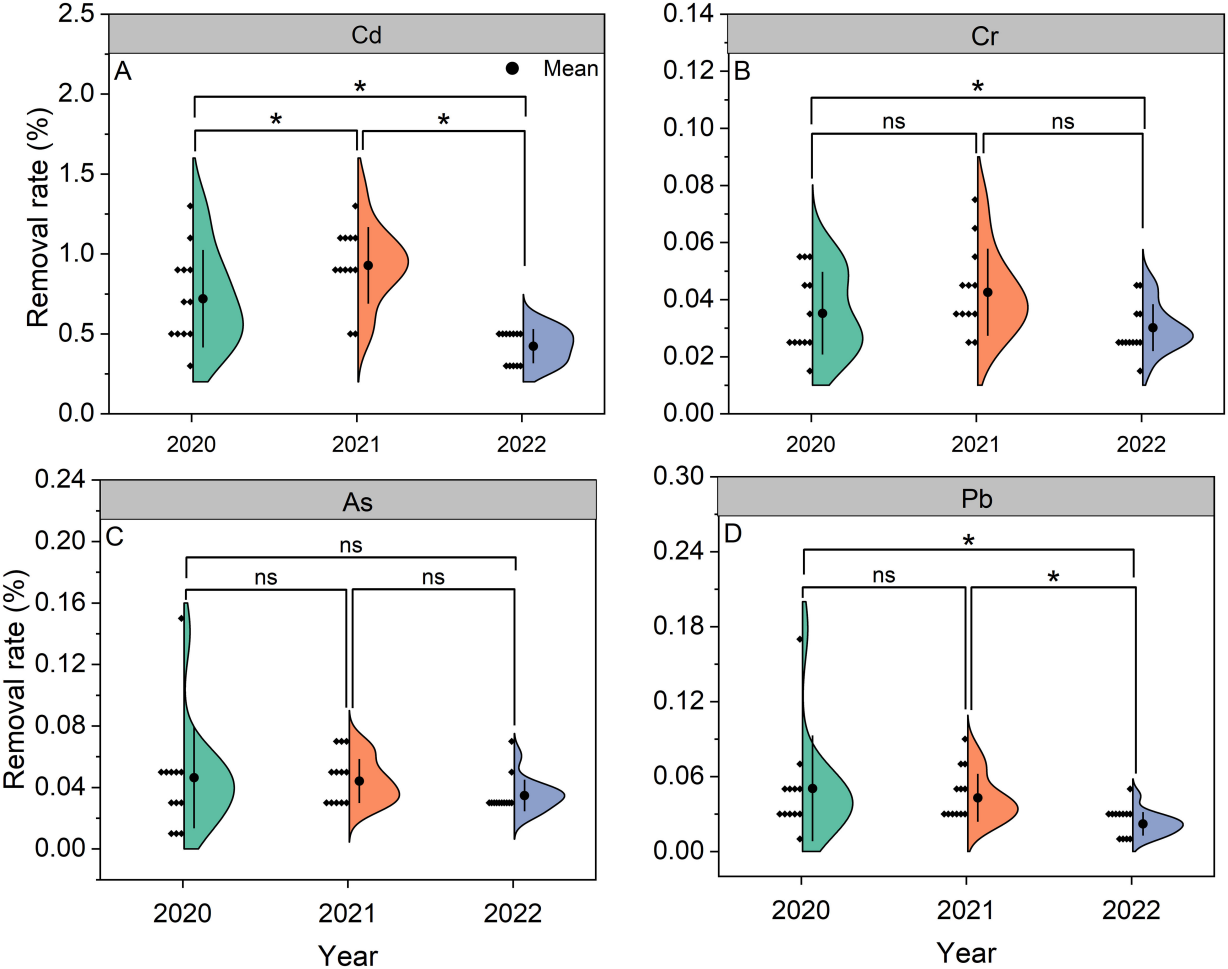
Removal rates of Cd **(A)**, Cr **(B)**, As **(C)**, and Pb **(D)** in *Pueraria thomsonii* during 2020–2022. Solid dots represent the means of 12 replicates. *denotes significance at the 0.05 probability level; ^ns^denotes non-significance at the 0.05 probability level.

Removal rates for Cd, Cr, As, and Pb ranged from 0.42% to 0.93%, 0.03% to 0.04%, 0.03% to 0.05%, and 0.02% to 0.05%, respectively, across 2020–2022, with the means of 0.69%, 0.04%, 0.04%, and 0.04% (**Figure 5**). The Cd removal rates in 2020 and 2022 were lower than in 2021 by 22% and 54%. Compared to 2021, the Cr removal rate in 2022 decreased by 29%. No significant interannual differences occurred for As removal rates. Consistently, the Pb removal rate in 2022 showed reductions of 56% and 49% relative to 2020 and 2021.

#### 3.4.1 Quantitative impact of soil Cd pollution threshold on remediation efficiency

**Figure 6:**
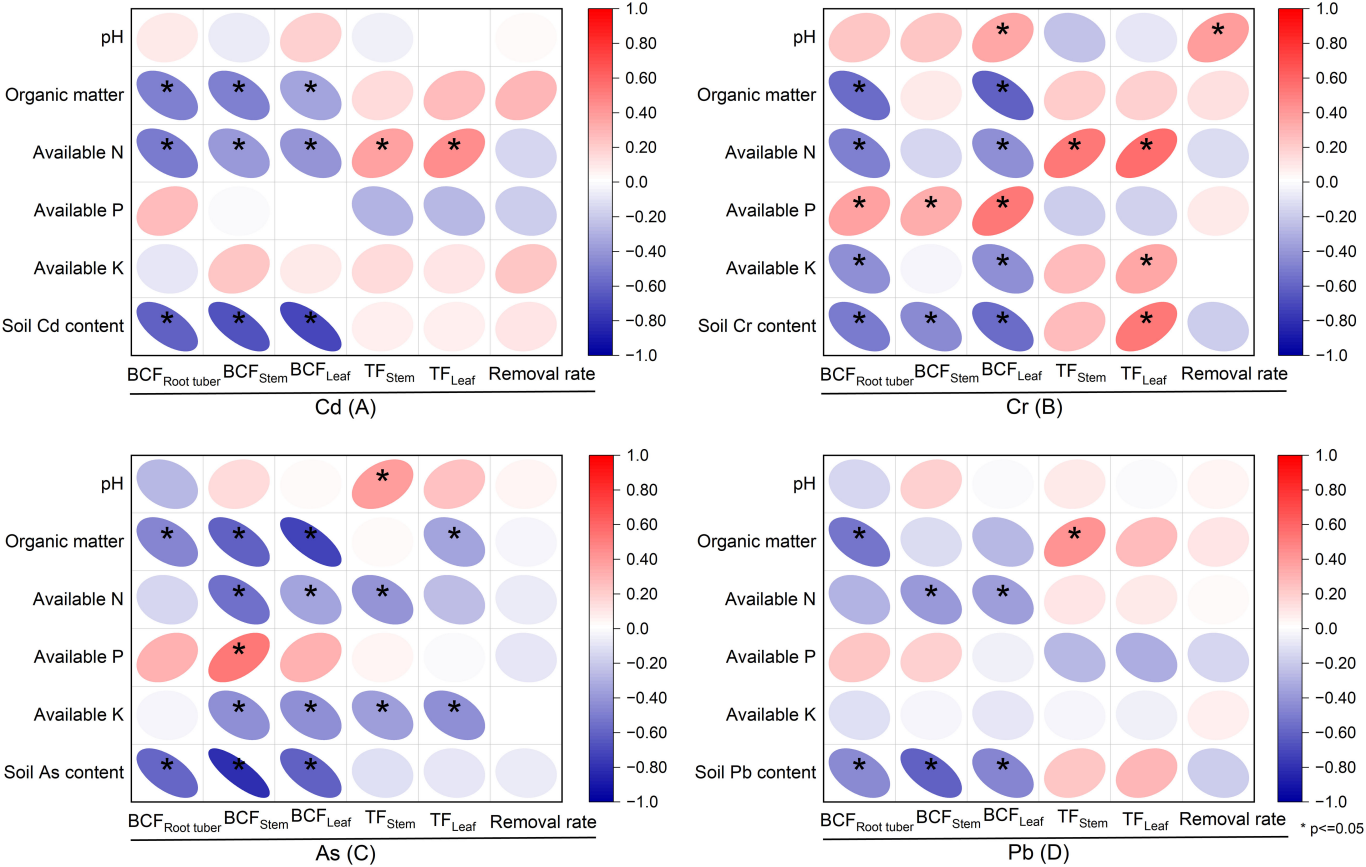
Relationships of soil properties to bioconcentration factors (BCFs), translocation factors (TFs), and removal rates in *Pueraria thomsonii* during 2020–2022 (*n* = 36). Data are the 36 replications in 3 years. * denotes significance at the 0.05 probability level.

To elucidate the quantitative relationship between soil Cd contamination levels and the remediation efficiency of *P. thomsonii*, we analyzed the correlation between soil Cd content and key remediation indicators, including total Cd accumulation in plants and Cd removal rate. The results demonstrated a significant positive correlation between soil Cd content and total Cd accumulation in plants at MA (*R*² = 0.16, *P* < 0.05; **Figure 4A**). Specifically, as soil Cd increased from 6.05 to 23.57 mg kg^−1^, total Cd accumulation rose from 53.67 to 298.73 g ha^−1^, while removal rates varied between 0.26% and 1.40%. These findings indicate that *P. thomsonii* maintains effective Cd uptake across a range of contamination levels, with higher soil Cd content leading to greater absolute extraction, albeit with a non-linear response in removal rates due to physiological saturation and soil–metal interactions (*P* > 0.05; **Figure 6A**).

### 3.5 Relationships of soil properties to bioconcentration factor, translocation factor, and removal rate

Significant negative correlations were observed between soil content of Cd, Cr, As, and Pb and their respective BCF values in root tubers, stems, and leaves (*P* < 0.05; **Figures 6A–D**). However, soil Cr content showed a positive correlation with leaf TF of Cr (*P* < 0.05; **Figure 6B**). For Cd, organic matter and available N were negatively correlated with the BCF in root tubers, stems, and leaves (*P* < 0.05). Conversely, available N also showed a positive correlation with stem and leaf TF of Cd (*P* < 0.05; **Figure 6A**). No significant correlation was observed between soil pH and the BCFs and TFs in root tubers, stems, and leaves for Cd. Regarding Cr and As, leaf BCF showed a negative correlation with organic matter, available N, and available K, but a positive correlation with available P (*P* < 0.05; **Figures 6B, C**). Available N and available K were positively correlated with leaf TF of Cr (*P* < 0.05; **Figure 6B**). Leaf TF of As showed a negative correlation with organic matter and available K (*P* < 0.05; **Figure 6C**). Leaf BCF of Pb was negatively correlated with available N (*P* < 0.05; **Figure 6D**).

## 4 Discussion

Plant species suitable for phytoextraction require rapid growth rates coupled with high-biomass production (Ali et al., 2013; Lima et al., 2019). This study corroborates such requirements, with *P. thomsonii* achieving a total biomass production of 17.95–21.22 t ha^−1^ during 2020–2021. However, total biomass production decreased significantly to 10.81 t ha^−1^ in 2022. This reduction is likely attributable to severe drought stress, as evidenced by a pronounced rainfall deficit throughout the critical tuber expansion period (approximately 100–225 days after sowing) in 2022 (**Figure 1C**), which consequently impaired normal plant development. Heavy metals disrupt key physiological processes in plants and induce phytotoxicity (Devi et al., 2022; Islam and Sandhi, 2023). In this study, the experimental field soil was confirmed to exhibit Cd contamination according to China’s national standard limits (GB 15618-2018) during 2020–2022, whereas Cr, As, and Pb concentrations remained within permissible thresholds. Therefore, *P. thomsonii* exhibited high-biomass production without Cd phytotoxicity symptoms (leaf chlorosis or necrosis) under severe Cd contamination (soil: 11.64–15.63 mg kg^−1^), indicating exceptional Cd tolerance.

In this study, root tuber concentrations of Cd, Cr, As, and Pb decreased significantly from SN to MA from 2020 to 2022. These results suggest that accelerated root growth drove substantial dry matter production. Although Cd uptake increased, its concentration decreased due to a typical dilution effect. This phenomenon was also observed in rice, which showed that the decrease of Cd accumulation in roots and shoots could be attributed to the dilution effect of increasing biomass (Cai et al., 2019). Root tuber Cd concentrations at MA in 2020, 2021, and 2022 were still higher than the medicinal plant safety threshold (0.30 mg kg^−1^). Although the root tuber of *P. thomsonii* does not meet hyperaccumulator criteria (Shao et al., 2017), it demonstrates significant phytoremediation potential for Cd-contaminated soils, achieving accumulation of 76.95–170.61 g ha^−1^ and Cd removal rates of 0.42%–0.93%. After undergoing multiple processes including cleaning and peeling, crushing and filtering, sedimentation and separation, and drying, the Cd content in the kudzu powder produced from *P. thomsonii* is significantly lower than the medicinal plant safety threshold (Lin et al., 2025). Otherwise, we found that *P. thomsonii* does not meet the strict concentration threshold (stems and leaves < 100 mg kg^−1^ for Cd). However, we argue that its exceptional combination of high biomass and effective Cd enrichment and translocation (BCF and TF > 1), resulting in a high Cd removal rate, makes it a highly promising high-biomass phytoremediation candidate, particularly for large-scale, sustainable cleanup of moderately Cd-contaminated farmlands where high biomass is as critical as metal concentration. The significant positive correlation between soil Cd content and Cd accumulation in plants further validates this practical utility. Consequently, future efforts should prioritize reducing Cd accumulation in root tubers or enhancing Cd translocation to shoots through molecular breeding approaches or optimized agronomic practices. Leaf and stem Cd concentrations showed no significant differences between EP and MA, while stem biomass consistently exceeded leaf biomass in this study. This indicates preferential Cd translocation from roots to stems for accumulation in *P. thomsonii*, evidenced by significantly higher Cd accumulation in stems versus root tubers and leaves at MA. Conversely, rice exhibits contrasting behavior, i.e., Cd was predominantly restricted to roots with minimal shoot translocation (Chiao et al., 2019). The possible reason for the difference between this study and the previous study is the different cultivation environment. Specifically, paddy flooding promotes iron plaque-mediated Cd sequestration in rice roots through *OsHMA3* suppression (Yan et al., 2016; Cao et al., 2018), whereas in aerobic farmland conditions of *P. thomsonii* cultivation, enhanced Cd accumulation in stems may stem from greater xylem loading capacity coupled with elevated transpiration rates acting as the primary driving force (Uraguchi et al., 2009). Leaf Cr, Pb, and As concentrations were significantly lower at EP than at MA. These results indicate that Cr, As, and Pb predominantly accumulated in foliar tissues of *P. thomsonii*. This is also supported by the results of this study showing elevated Cr, As, and Pb accumulation in the leaves relative to root tubers and stems at MA. In addition, an intriguing discovery was that Cd accumulation rates in *P. thomsonii* were significantly higher during SN to EP than during EP to MA. Conversely, Pb exhibited the reverse temporal pattern. Cr and As accumulation rates showed no significant differences between the two periods. This difference may stem from distinct physiological strategies: Cd^2+^ likely utilizes early-stage Ca^2+^ channels during rapid growth, while Pb^2+^ accumulation relies on late-stage chelator synthesis (Ruley et al., 2006; Mei et al., 2014). Cr and As accumulation remained stable, indicating that constitutive translocation mechanisms were less affected by developmental cues.

Our results underscore a clear quantitative relationship between soil Cd pollution thresholds and the remediation efficiency of *P. thomsonii*. The strong positive correlation between soil Cd and plant accumulation confirms its suitability for moderately to highly contaminated soils. The non-linear response in removal rate, however, suggests the existence of physiological saturation points or modulation by soil properties. This implies that *P. thomsonii* can provide a consistent, though not proportionally increasing, removal service in soils with high Cd content. Its high biomass and perennial nature thus position it as a viable candidate for long-term remediation strategies in such environments.

The BCF, characterizing a plant’s absorption, tissue mobilization, and storage of soil elements, and TF, a key criterion widely studied for assessing element hyperaccumulation, are both important metrics (Mu et al., 2019; Seridou et al., 2025). For hyperaccumulator classification, both BCF and TF values must exceed 1.0 (Liu et al., 2016). In this study, stem and leaf Cd BCF values ranged from 1.09 to 1.34, while TF values ranged from 4.28 to 9.10, demonstrating *P. thomsonii*’s remarkable capacity for Cd enrichment and efficient transport. These findings align with the result of Lin et al (Lin et al., 2025), who conducted a field experiment in three sites with Cd-, As-, and Pb-contaminated soils, where both Cd-BCF and Cd-TF values in all plant tissues consistently exceeded 1.0. In addition, BCF values for Cr, As, and Pb in root tubers, stems, and leaves remained below 1.0, while TF values exceeded 1.0 in all cases except stem Cr-TF during the 2022 growing season in this study. The low BCF values suggest that these metals are less readily absorbed by the plant, potentially due to lower uptake efficiency or limited mobility within plant tissues. This variation in metal enrichment may be linked to the plant’s selective ion transport mechanisms and the differing affinities of these metals for specific transporters (Millaleo et al., 2010). Conversely, the high TF values indicate efficient root-to-shoot transport of heavy metals in *P. thomsonii*, possibly driven by enhanced xylem loading capacity, which facilitates upward metal mobilization (Uraguchi et al., 2009). Soil properties had a strong influence on the BCF and TF of heavy metals (Al-Huqail et al., 2025). In this study, soil Cd, Cr, As, and Pb concentrations showed significant negative correlations with their respective BCF values in root tubers, stems, and leaves. This suggests that elevated soil metal levels likely suppress plant metal accumulation due to phytotoxicity effects. Additionally, the observed negative correlation between heavy metal BCFs and soil organic matter, consistent with the findings of Zeng et al (Zeng et al., 2011), could be attributed to the ability of organic matter to adsorb heavy metals or form stable complexes with humic substances, thereby reducing their bioavailability in the soil. However, Deepika and Haritash (Deepika. and Haritash, 2023) reported a negative correlation between soil pH and BCF, contrary to our findings of no significant correlation. This divergence may stem from limited pH variability. Specifically, the pH range across the 3-year field experiment was narrow (5.64–5.87), as shown in **Table 1**. Within this consistently acidic and relatively stable range, the solubility and bioavailability of Cd were likely already high and not the primary limiting factor for plant uptake. Consequently, the subtle year-to-year pH fluctuations were overshadowed by the variations in other soil properties, such as organic matter and available nitrogen, which exhibited stronger correlations with BCF and TF. Therefore, further investigation is required to elucidate the underlying drivers of these conflicting results. Available N showed a positive correlation with the TF of Cd and Cr, whereas available K exhibited a negative correlation with the TF of As. Notably, neither available N nor K demonstrated significant correlations with the TF of Pb. These differential relationships likely arise from element-specific transport mechanisms and the low bioavailability of Pb, dominated by soil adsorption rather than nutrient interactions.

A critical consideration for the safe application of *P. thomsonii* in Cd-contaminated farmland is the potential risk of Cd transfer from accumulated organs (stems and leaves) to the edible root tubers. Our results clearly demonstrate that Cd is preferentially allocated to the stems rather than the root tubers at maturity. The high TFs from root tubers to stems and leaves (**Table 4**) indicate that the movement of Cd is predominantly upward via the xylem, driven by transpiration, with minimal retranslocation back to the storage root. This physiological partitioning effectively reduces the risk of Cd migration into the edible part. Furthermore, post-harvest processing such as peeling and washing of root tubers significantly reduces the final Cd content in kudzu powder, ensuring it remains below safety thresholds. Therefore, the risk of Cd contamination in the edible product from stem or leaf sources is considered low.

It is noteworthy that *P. thomsonii* straw and waste residues, which contain substantial Cd, pose risks of secondary pollution. Practical mitigation and valorization strategies include the following: 1) phytosmelt extraction of straw: This technology involves the thermal conversion of biomass into a “bio-ore” for subsequent metal recovery. While highly effective in concentrating and recovering metals, its current application may be limited to centralized facilities or high-value scenarios due to considerable operational costs and energy demands. 2) Recycling into cardboard materials: This approach sequesters the Cd within the product matrix, providing a cost-effective and easily implementable disposal route. 3) Field application of bio-fermented straw as a low-Cd fertilizer: During anaerobic fermentation, Cd can be immobilized into stable complexes (e.g., with sulfides) or tightly incorporated into organic matter, significantly reducing its bioavailability (Stevenson et al., 2017). 4) Utilizing high-Cd load root tubers for industrial alcohol production: The fermentation and distillation process can yield Cd-free ethanol, while concentrating Cd in the residual mash, which can then be managed centrally.

## 5 Conclusions

*Pueraria thomsonii* exhibited high-biomass production (10.81–21.22 t ha^−1^) during 2020–2022. Cd demonstrated superior remediation performance relative to Cr, As, and Pb, with significantly higher total accumulation and soil removal rates. Critically, stems served as the primary sink for Cd, whereas Cr, As, and Pb predominantly accumulated in the leaves. The stem and leaf BCF and TF values of Cd consistently exceeded 1. These findings indicate that the combination of high-biomass production and elevated Cd accumulation in aerial tissues positions *P. thomsonii* as a promising candidate for high-biomass phytoremediation of Cd-contaminated farmland soils.

## Data Availability

The original contributions presented in the study are included in the article/supplementary material. Further inquiries can be directed to the corresponding author.
